# Proteome-wide analysis reveals widespread lysine acetylation of major protein complexes in the malaria parasite

**DOI:** 10.1038/srep19722

**Published:** 2016-01-27

**Authors:** Simon A. Cobbold, Joana M. Santos, Alejandro Ochoa, David H. Perlman, Manuel Llinás

**Affiliations:** 1 Lewis-Sigler Institute for Integrative Genomics, Princeton University, Princeton, NJ 08544; 2 Center for Statistics and Machine Learning, Princeton University, Princeton, NJ 08544; 3 Department of Molecular Biology, Princeton University, Princeton, NJ 08544; 4 Department of Chemistry Princeton University, Princeton, NJ 08544; 5 Collaborative Proteomics and Mass Spectrometry Center, Princeton University, Princeton, NJ 08544; 6 Department of Biochemistry and Molecular Biology, Department of Chemistry, Center for Malaria Research and Center for Infectious Disease Dynamics, W126 Millennium Science Complex, Pennsylvania State University, State College, PA 16802, USA

## Abstract

Lysine acetylation is a ubiquitous post-translational modification in many organisms including the malaria parasite *Plasmodium falciparum*, yet the full extent of acetylation across the parasite proteome remains unresolved. Moreover, the functional significance of acetylation or how specific acetyl-lysine sites are regulated is largely unknown. Here we report a seven-fold expansion of the known parasite ‘acetylome’, characterizing 2,876 acetylation sites on 1,146 proteins. We observe that lysine acetylation targets a diverse range of protein complexes and is particularly enriched within the Apicomplexan AP2 (ApiAP2) DNA-binding protein family. Using quantitative proteomics we determined that artificial perturbation of the acetate/acetyl-CoA balance alters the acetyl-lysine occupancy of several ApiAP2 DNA-binding proteins and related transcriptional proteins. This metabolic signaling could mediate significant downstream transcriptional responses, as we show that acetylation of an ApiAP2 DNA-binding domain ablates its DNA-binding propensity. Lastly, we investigated the acetyl-lysine targets of each class of lysine deacetylase in order to begin to explore how each class of enzyme contributes to regulating the *P. falciparum* acetylome.

Lysine acetylation is a protein post-translational modification (PTM) that significantly alters the biochemistry of lysine residues and is known to confer considerable changes to protein function. The emergence of lysine acetylation as a PTM that is widespread and conserved across proteomes from prokaryotes to mammals has highlighted the importance of this modification beyond its long-appreciated role in histone modification^1^,^2^,^3^. Despite the importance of lysine acetylation to protein function and cellular regulation, it was only recently established that acetylation of non-histone proteins was present in *Plasmodium falciparum*^4^, the parasite most responsible for severe and fatal malaria. *P. falciparum* possesses four lysine acetyltransferases (KATs, which acetylate specific lysine residues)^5^,^6^ and five lysine deacetylases (KDACs) which consist of one class I, two class II and two NAD-dependent class III (sirtuins). Previous studies have implicated the sirtuins Sir2A and Sir2B in antigenic variation through histone deacetylation, but whether these proteins or the class I and II KDACs act on other acetylated (non-histone) proteins has not been established^7^,^8^,^9^.

Malaria remains a substantial burden on global health and the emergence of resistance to nearly all available antimalarials makes treatment in endemic countries increasingly difficult. Research towards identifying novel antimalarial targets is therefore essential to confronting this global problem. The importance of histone acetylation for cellular development has prioritized the KATs and KDACs as attractive candidates for antimalarial research, although the antimalarial potential of targeting the regulators of acetylation may exceed histone acetylation alone. To date, several studies have investigated exploiting parasite KDACs as novel antimalarial targets^10^,^11^,^12^,^13^,^14^. However, the exploration of KDACs as potential new antimalarial targets requires the characterization of each enzyme’s substrate specificity and the functional relevance of their deacetylase activity to parasite development.

Here we describe an expansion in the known *P. falciparum* acetylome by seven-fold, characterizing 2877 sites on 1146 proteins by mass spectrometry (MS). We find acetylated proteins to be present in all major compartments of the infected erythrocyte with acetyl-lysine especially prevalent on metabolic and transcription-associated proteins. Using stable isotope labeling with amino acids in cell culture (SILAC) and quantitative mass spectrometry-based proteomics, we demonstrate that inhibition of class I and II KDACs increases protein acetylation of chromatin-remodeling proteins. Interestingly, we observe acetylation of transcriptional proteins to be dependent upon acetyl-CoA metabolism, whereby changes in the acetate/acetyl-CoA balance result in increased acetylation of several ApiAP2 DNA-binding proteins. These findings suggest acetylation may play a more complex role in transcription (beyond histones), as we show that acetylation of a specific lysine within one of the three DNA-binding domains of the ApiAP2 protein PF3D7_1007700 leads to a loss in DNA-binding. These results reveal the breadth of acetylation of the *Plasmodium* proteome and indicate that the parasite’s transcriptional program may be mediated in part by metabolic signaling and acetylation.

## Results

### Lysine acetylation in *Plasmodium falciparum*

To generate high quality *P. falciparum* protein extracts for analysis by mass spectrometry, synchronous parasite-infected erythrocytes were grown to the trophozoite stage before parasite release by saponin treatment. ‘Isolated’ parasites were washed and lysed directly in guanidine lysis buffer and the protein solution was subjected to large-scale filter-aided sample preparation (FASP) digestion^15^, followed by strong cationic exchange to reduce sample complexity and improve detection of low abundant acetylated peptides. A portion of the fractionated peptide pools were subjected to enrichment by immunoprecipitation using anti-acetylated lysine antibodies, thereby partitioning the samples further into enriched and unenriched fractions. Finally, all peptide samples were analyzed by nano-flow ultra-high performance liquid chromatography (nano-UPLC) tandem mass spectrometry (MS/MS), followed by database search against the *P. falciparum* and human erythrocyte proteomes. From over 3.5 million tandem mass spectra (3,624,625), nearly half (1,620,607) resulted in high-confidence peptide spectral matches (PSMs) within a 1% false discovery rate. In total, we obtained 17,870 PSMs to acetylated peptides, which correspond to 2,876 distinct lysine acetylation sites on 1,143 proteins after coalescence of redundant site identifications from repeat and overlapping peptides (Supplemental Table 1). The number of acetylated peptides characterized in the anti-acetyl lysine antibody enriched and un-enriched fractions were 1,157 and 2,148, respectively, with 729 identified in both fractions.

Overall our expanded dataset is in good agreement with Miao *et al.* 2013, with 40% (168/421) of the previously reported acetyl-lysine sites also detected in this study (Supplemental Table 2), consistent with the different methodological approaches taken. Furthermore we detected 29 of the 34 previously identified histone acetylation sites^4^,^16^, providing a second validation of the robustness of the acetyl-lysine assignments. In addition, we characterized 24 novel histone acetylation sites in *P. falciparum*, expanding the map of this modification to 58 sites across all eight histones (Supplemental Fig. 1A). Given the large number of novel histone acetylation sites detected, we assessed the conservation across disparate eukaryotic organisms using the Compendium of Protein Lysine Modifications database^17^. In instances where the histone sequences are conserved between *P. falciparum*, *S. cerevisiae*, *D. melanogaster*, *M. musculus* or *H. sapiens*, lysine acetylation is remarkably well conserved across organisms, with 41/43 sites identified in *P. falciparum* present in at least one other organism (Supplemental Fig. 1B).

### Compartmental distribution and enrichment analysis of acetylated proteins and sites

**Figure 1:**
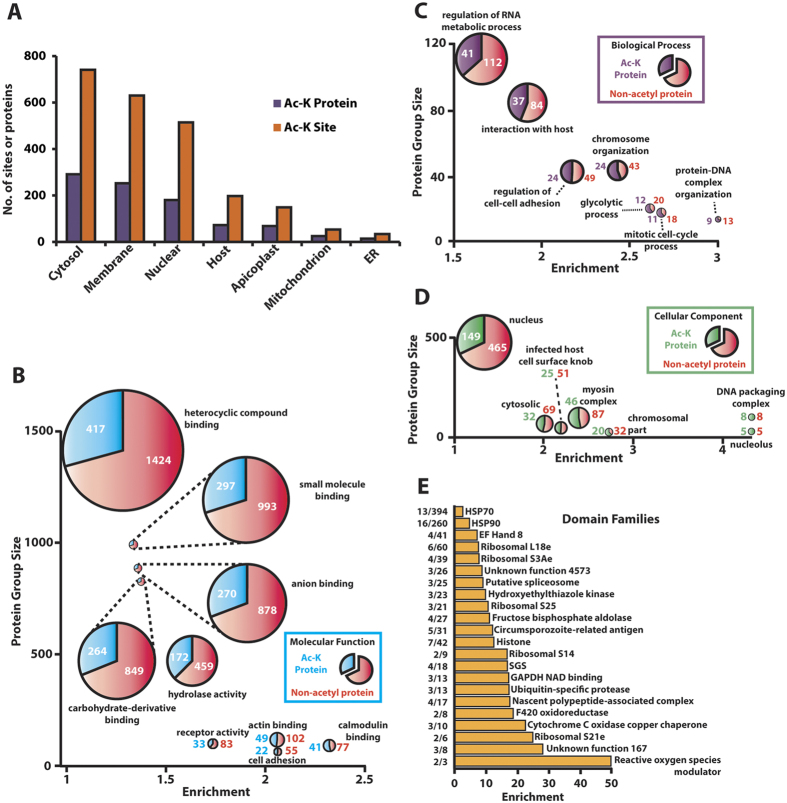
(**A**) Distribution of acetylated protein and acetyl-lysine sites across subcellular compartments. Predicted localization of acetylated proteins from annotated GO terms. Enrichment of (**B**) molecular function (**C**) biological process (**D**) cellular component GO terms for acetylated proteins. Statistically significant enrichment of 22 terms across the three GO term groups was determined using hypergeometric testing (*q* < 0.01). 5 redundant groups were removed for clarity. (**E**) Acetyl-lysine enrichment of protein domains was assessed using three different approaches (see methods). Presented are the protein domains significantly enriched for acetylated lysines using the tiered stratified q-value method. Pfam families are listed as significantly enriched if their E-value is <1. The y-axis represents a ratio of the lysines present in each domain family acetylated to the total number of lysines within the domain family.

To assess the subcellular distribution of the acetylated proteins, we examined the enrichment of Gene Ontology (GO) term annotations associated with these proteins (Fig. 1A). The acetylome contains proteins known to be found across all major subcellular compartments, particularly the cytosol, membrane and nucleus. We therefore assessed the known functional annotations of the acetylated proteins to predict how acetylation might contribute to parasite development. Twenty-three non-redundant GO terms were statistically enriched for acetylated proteins (*q* < 0.01) covering molecular function, biological processes and cellular component (Fig. 1B–D; Supplemental Table 3). These included metabolic enzymes, represented by both the carbohydrate-derivative binding term (molecular function) and glycolytic process (biological process). Proteins associated with chromosome organization and regulating RNA metabolic processes were also notable for their acetyl-lysine enrichment (biological process).

**Figure 2:**
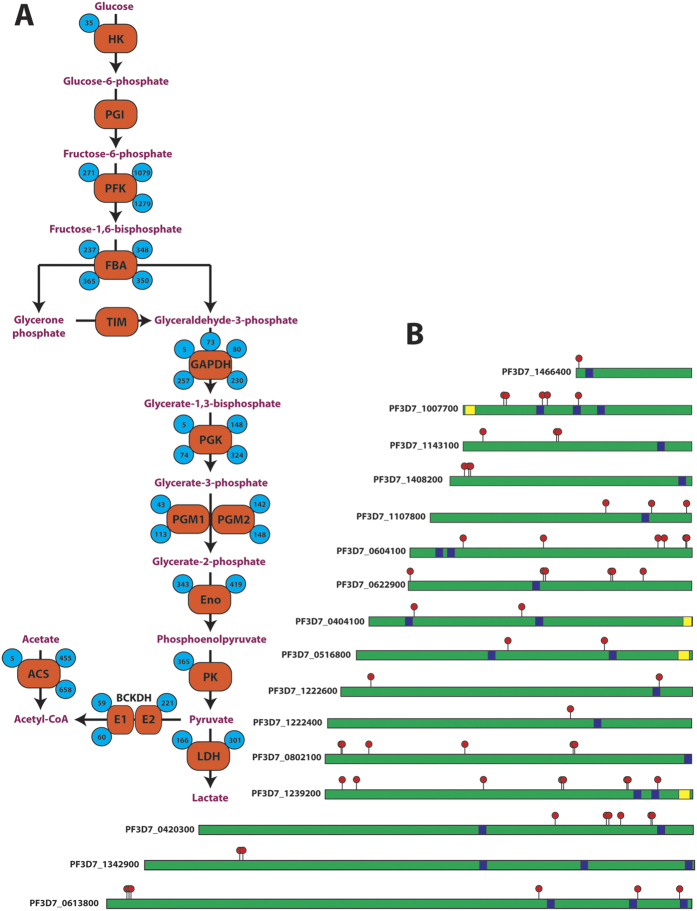
(**A**) Acetylation of glycolytic and acetyl-CoA biosynthetic enzymes. Numbers inside blue circles indicate the position of the acetyl-lysine site, enzymes represented are hexokinase (HK), phosphoglucose isomerase (PGI), phosphofructokinase (PFK), fructose bisphosphate aldolase (FBA), triose phosphate isomerase (TIM), glyceraldehyde-3-phosphate dehydrogenase (GAPDH), phosphoglycerate kinase (PGK), phosphoglycerate mutase 1/2 (PGM1/2), enolase (Eno), pyruvate kinase 1 (PK), lactate dehydrogenase (LDH), branched-chain keto-acid dehydrogenase subunit E1/E2 (BCKDH E1/E2), acetyl-CoA synthetase (ACS). (**B**) Distribution of acetyl-lysine on ApiAP2 DNA-binding proteins. Red flags indicate acetylated lysines, blue boxes represent AP2 DNA-binding domains and yellow boxes represent ACDC domains (AP2-coincident domain mostly at the C-terminus). The non-represented ApiAP2 proteins are not acetylated.

To probe the possible functional consequences of acetylation on the diversity of proteins identified in the acetylome, we determined the functional enrichment of individual protein domains (Fig. 1E). 22 domain families were enriched for acetylated lysines and were predominantly composed of ribosomal and metabolic proteins (Supplemental Table 3). Of particular note, ribosomal proteins are extensively acetylated and form an integrated network with other translation-related proteins (Supplemental Fig. 2). Moreover, the significant enrichment for acetylation in glycolytic enzymes (Figs 1C and 2A) and within glycolytic domains (Fig. 1E) may suggest a role for acetylation in modulating glycolysis, as observed in other organisms^3^. Interestingly, ApiAP2 DNA-binding proteins are extensively acetylated as well (Fig. 2B), with 16 (out of 27) members possessing 62 acetyl-lysine sites.

**Figure 3:**
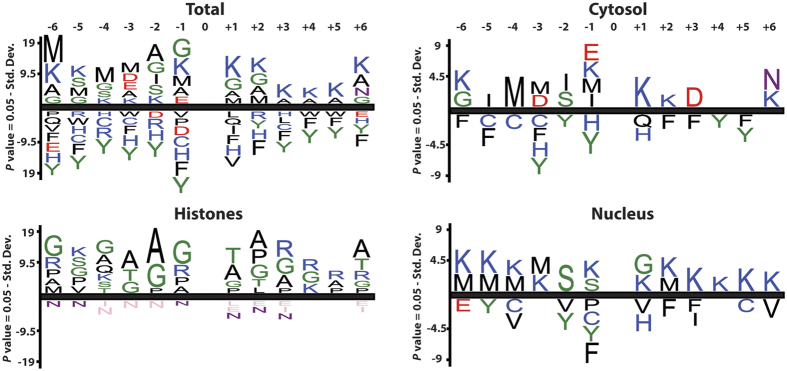
Sequence motifs surrounding acetylated lysines. Sequence motif surrounding lysine acetylation presented as significantly enriched/de-enriched amino acids at positions −6 through +6 as percentage difference (*p* < 0.05).

To determine whether lysine acetylation in *P. falciparum* follows an amino acid sequence preference, we analyzed the sequence motif surrounding acetylated lysines in our dataset. Globally, we observed that the aromatic residues phenylalanine and tyrosine were significantly de-enriched neighboring acetylated lysines (Fig. 3). In contrast, the small neutral amino acids glycine and alanine were significantly enriched (along with other lysines). Compartment-specific analysis of acetylation site sequence motifs indicated that cytosolic sites had a significant enrichment for glutamate at the -1 position, whereas nuclear sites (excluding histones) were strongly enriched for lysine across the entire motif, indicating some compartmental acetyl-lysine specificity (Fig. 3).

While the primary focus of this study was to investigate parasite-specific acetylation, the depth of our MS interrogation of the samples and high sensitivity of MS detection also enabled the identification of 197 acetylation sites on 72 *P. falciparum* proteins previously annotated as exported outside of the malaria parasite and 247 acetylation sites on 87 host erythrocyte proteins (Supplemental Table 4), which remained present in our samples in low abundance post-saponin lysis and parasite isolation. These results suggest that acetylation is a relatively abundant modification in the infected host compartment as well. Human erythrocytes possess both SIRT2 and SIRT5 sirtuin proteins which are likely responsible for mediating most acetylation in the host compartment^18^, yet it remains an open question whether the parasite modulates acetylation of host proteins, alters their abundances, or whether parasite-exported proteins are acetylated in the host compartment by host enzymes.

### Dynamic changes in acetylation following KDAC inhibition or genetic disruption

**Figure 4:**
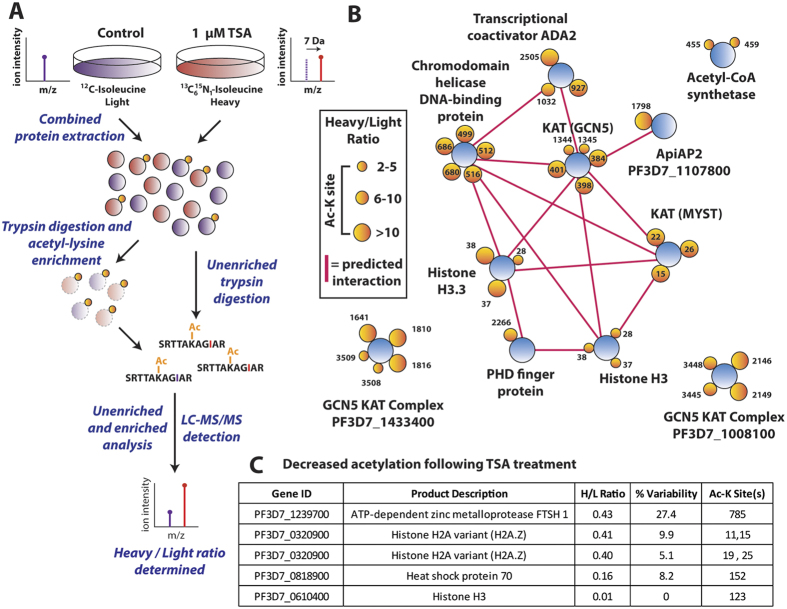
(**A**) Stable isotope labeling by amino acids in cell culture. *P. falciparum* infected erythrocytes are maintained under standard culturing conditions in RPMI containing either ^12^C_6_^14^N_1_ isoleucine (light) or ^13^C_6_^15^N_1_ isoleucine (heavy) for 72 hours. After proteome labeling (>95%), the light culture was untreated and the heavy culture was treated with 1 μM of tricostatin A (TSA) for three hours. Treated (heavy) and untreated (light) cultures were saponin-permeabilized, washed, combined and extracted. Following trypsin digestion and SCX fractionation, samples were analyzed both directly (unenriched) and subsequent to enrichment by anti-acetyl lysine immunoprecipitation via high resolution nano-UPLC-MS and MS/MS. Relative ratios of the isoleucine bearing heavy (+7 Da) peptides to their light counterparts were measured to determine the ratios of acetylation in the original experiments. (**B**) Proteins containing the acetyl-lysine sites that were quantified as undergoing a >2-fold increase (heavy/light ratio >2) when treated with TSA. Proteins are represented as blue circles, individual acetyl-lysine sites as orange circles (with the residue position defined alongside/within). The size of each acetyl-lysine site is proportional to the increase in acetylation following TSA treatment (quantified as the treated/untreated heavy/light ratio). Network analysis was performed on the entire dataset to represent functionally-related proteins. Direct (physical) or indirect (functional) interactions were defined with experimental, databases and text-mining information sources using STRING 9.1 with a medium confidence cutoff (0.4) and are represented by a pink line between proteins. (**C**) A subset of acetyl-lysine sites that decreased following TSA treatment (<0.5 heavy/light ratio). Percent variability is a measure of the heavy/light ratio variation between repeated measurements, where a value of 0 represents repeated measurement at the extreme minimum quantification range. The Ac-K site indicates the lysine residue position that underwent decreased acetylation. Supplemental Table 5 and 6 contain the complete list of altered acetylation sites under TSA treatment.

Prioritizing KDACs for further interrogation as candidate drug targets requires a complete understanding of the acetylated-lysine targets of each KDAC. The parasite class I and II KDACs are zinc-dependent enzymes that release acetate during lysine deacetylation^19^. In contrast, the deacetylase activity of class III sirtuins is functionally distinct, requiring NAD as a cofactor and generating O-acetyl-ADP-ribose during lysine deacetylation. By using the class I and II KDAC inhibitor, tricostatin A (TSA), in a quantitative proteomics approach, we analyzed which acetylated lysines are targeted by the class I and II KDACs. Conventional stable isotope labeling with amino acid in cell culture (SILAC), which uses isotopically-labelled arginine and/or lysine, is not feasible in *P. falciparum* because of the constant supply of unlabeled amino acids generated via hemoglobin catabolism. However, the parasite has an exogenous requirement for isoleucine^20^, making it possible to use ^13^C_6_^15^N_1_ isoleucine to fully label the parasite proteome and enable quantitative measurements by MS (Fig. 4A)^21^. H/L measurements were stringently filtered based on the number of peptide spectral matches leading to the peptide identification and the percent variability of quantification (see methods) to improve the accuracy of quantification. However, we have used network analysis to focus on changes in acetylation across functionally-associated proteins and thereby increase confidence in the results observed.

Following ^13^C_6_^15^N_1_ isoleucine proteome labeling, a parasite culture was treated with 1 μM of TSA for three hours. An identical culture was maintained under non-labeling conditions as control. After TSA exposure, parasites were saponin isolated, washed, combined and the protein extracted. The heavy (TSA treated) and light (untreated) isoleucine-containing acetylated peptides were differentiated by a mass shift of 7 Da, and the heavy-light ratio of these species was quantified. Inhibition of both class I and II KDACs increased acetylation greater than two-fold in 5.0% (≤1% FDR) of detected sites (Supplemental Fig. 3 and Supplemental Table 5 and 6). The class I and II deacetylases putatively target acetyl-lysine residues on acetyltransferases (MYST, GCN5 and GCN5 complex members), histones and transcription-associated proteins (Fig. 4B). The proteins that underwent increased acetylation following TSA treatment are functional or physical interaction partners, as indicated by a STRING9.1 network analysis. TSA treatment did not lead to a change in the total protein abundance, confirming that the changes in acetylation levels observed were genuine changes in acetylation site occupancy and not an indirect consequence of altered protein abundance following drug treatment (Supplemental Table 6).

The specificity of TSA towards inhibiting KDACs that target transcription-associated proteins may explain the previously observed changes in parasite transcription during TSA treatment^10^. Increased histone acetylation would typically lead to relaxed chromatin and increased transcription, yet Andrews *et al.* also observed a large number of transcripts decreasing in abundance following TSA treatment. It is plausible that the decrease in transcription following TSA treatment may be a result of altered acetylation of non-histone proteins that participate in transcriptional regulation. Interestingly, several histone lysine residues underwent decreased acetylation following TSA treatment (Fig. 4C), potentially indicating a feedback signaling response following inhibition of the class I/II KDACs.

We also measured changes in lysine acetylation in parasite lines possessing disrupted copies of the class III KDACs Sir2A and Sir2B using a similar isoleucine SILAC quantitative proteomics approach. Relative measurements of the acetylation state of Sir2A and Sir2B (heavy isoleucine) compared to their parental lines (light isoleucine) revealed that few changes had occurred (Table 1/Supplemental Table 7 and 8). Despite substantial quantification of acetylated peptide ratios between the Sir2A knockout and the parental line (817 H/L pairs) and the Sir2B knockout and the parent line (793 H/L pairs), only 14 acetylated peptides in the case of the Sir2A knockout and 13 in the Sir2B knockout showed a greater than two-fold change over the parent line (Supplemental Figs 4 and 5). Acetylation of histone H4 at position K80 was elevated in the Sir2A knockout line consistent with Sir2A mediating *var* gene repression via H4 deacetylation^8^,^9^,^22^,^23^,^24^. Sir2B has previously been characterized to regulate *var* gene expression via deacetylase activity^9^. Here we did not observe any change in the total (detectable) histone acetylation state following Sir2B disruption. One explanation is that the global histone proteomic analysis performed here obscured the detection of changes to histone acetylation localized to specific chromosomal regions. Alternatively, the peptide coverage across the histone component in our dataset available for SILAC quantification may not have been sufficient to capture the specific histone sites involved in the response to Sir2B disruption.

In comparison to chemical inhibition of the class I and II KDACs, genetic disruption of each sirtuin led to few changes in acetylation (compared to the wild-type parasite line) and may reflect a high level of redundancy between each sirtuin.

### The role of lysine acetylation in DNA-binding of an ApiAP2 protein

While the transcriptional program of *P. falciparum* during intraerythrocytic development exhibits a periodic cascade of gene expression, how the parasite regulates this ‘just-in-time’ transcriptional profile remains unknown^25^. The ApiAP2 family of DNA-binding proteins has recently emerged as a major class of transcription factors in *P. falciparum*, each possessing between one and three AP2 DNA-binding domains^26^,^27^,^28^. Each AP2 domain binds a specific DNA sequence motif, with some domains also capable of binding a secondary motif^26^. Given the large number of acetyl-lysine sites detected on AP2 DNA-binding proteins (Fig. 2B), we hypothesized that acetylation may play a role in transcriptional regulation by altering the binding and recruitment of ApiAP2 DNA-binding proteins.

**Figure 5:**
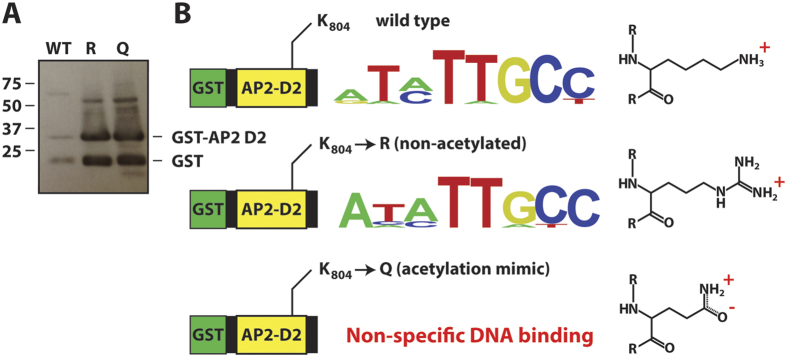
(**A**) Western blot of GST-fused AP2 binding domain 2 from PF3D7_1007700. The two mutant GST-fusion proteins (K804R and K804Q) were tested alongside the wild type version, which was used as control since it has been previously shown to specifically bind DNA^26^. (**B**) Protein binding microarrays of GST-D2 wt and GST-D2R mutant bind essentially the same DNA motif, while GST-D2Q mutant does not bind DNA in a sequence specific manner. An enrichment score cut-off of 0.45 was used to distinguish high affinity binding data from low affinity and non-specific binding.

The ApiAP2 DNA-binding protein PF3D7_1007700 (PF10_0075) possesses three AP2 DNA-binding domains, with domains 1 and 2 containing acetyl-lysine modifications (Fig. 2B). To mimic the effect of acetylation on the AP2 domains, we substituted K555 and K804 with either arginine or glutamine and expressed and purified these GST-tagged AP2 domains from *E. coli*. The arginine mutant simulates an unacetylated lysine, maintaining the positive charge on the side-chain; whereas glutamine closely resembles an acetylated-lysine, with a neutral medium-sized side-chain. Such mutations have been previously used to dissect the function of acetylation on specific lysine residues^29^. Although the mutant domain 1 fusion species were recalcitrant to purification, the mutant GST-PF3D7_1007700 AP2 domain 2 (GST-D2) was successfully purified (Fig. 5A) and its DNA-binding specificity analyzed by protein-binding microarray (PBM)^26^,^30^. Substitution of lysine to arginine (GST-D2Rmutant) resulted in no change in the bound DNA sequence motif compared to the wild type construct (GST-D2wt) (Fig. 5B), consistent with a requirement for maintaining the positive charge (lysine in a deacetylated state) for binding specificity. Conversely, loss of the positive charge through substituting lysine for glutamine (GST-D2Qmutant) resulted in a complete loss of DNA binding by the AP2 domain (Fig. 5B), suggestive of a similar effect in response to acetylation of these key lysine residues.

### Acetylation of transcription and translation-associated proteins is regulated by the acetate/acetyl-CoA balance

**Figure 6:**
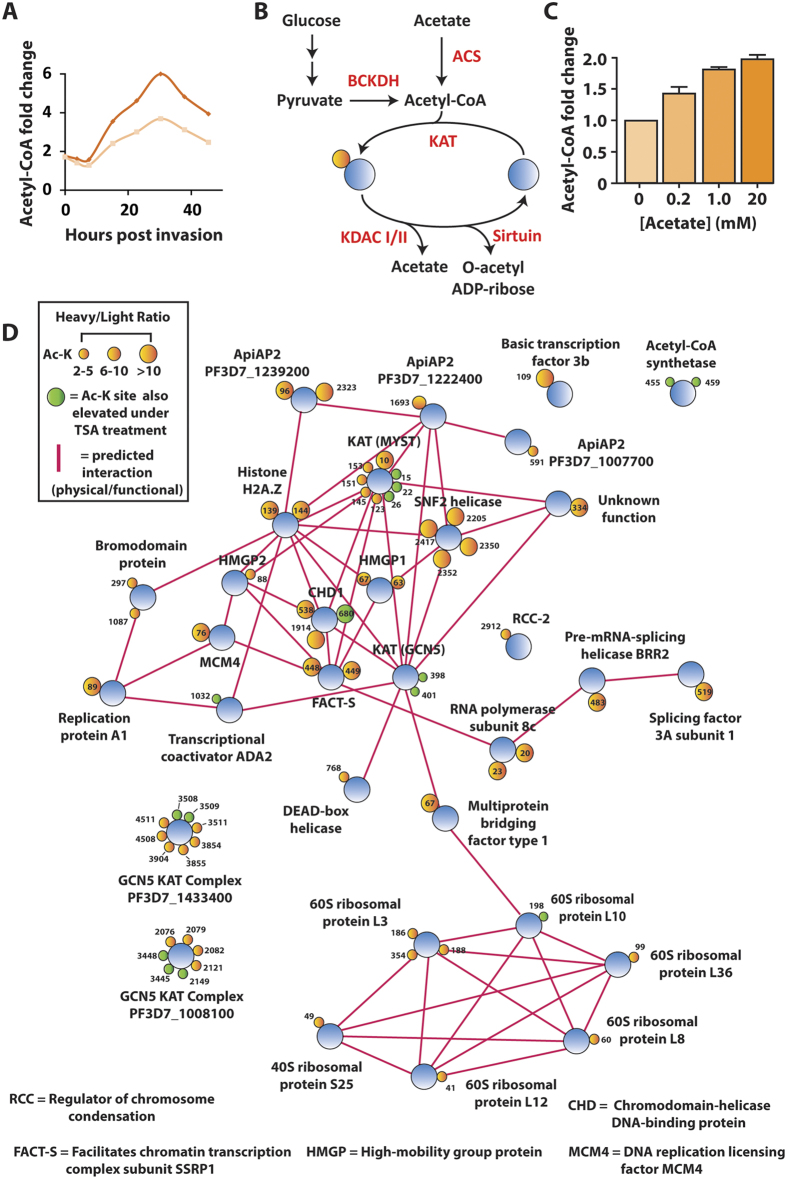
(**A**) The temporal profile of the acetyl-CoA pool in *P. falciparum*-infected erythrocytes across the intraerythrocytic developmental cycle. The data represents two independent time course experiments as the individual ion count of acetyl-CoA in infected erythrocytes relative to uninfected erythrocytes. These data were originally published in^60^ and have been reprocessed and normalized from the IG06 IDC to the standard 48 hour lifecycle of NF54. Each biological replicate is represented as a separate time series (orange and yellow lines). (**B**) Acetyl-CoA metabolism and acetylation in *P. falciparum*. The acetylation activity of acetyltransferases is dependent upon a supply of acetyl-CoA. (**C**) The intracellular pool of acetyl-CoA is influenced by the exogenous concentration of acetate. Enriched infected erythrocytes were incubated for one hour in RPMI containing increasing concentrations of acetate (pH adjusted to 7.4). The intracellular acetyl-CoA present in each condition was measured via LC-MS and presented as a fold-change above the control acetate-free condition (Av. ± SEM from n = 3). (**D**) Proteins containing the acetyl-lysine sites that were quantified as undergoing a >2-fold increase (heavy/light ratio >2) when treated with acetate (5 mM for 3 hours). Proteins are represented as blue circles, individual acetyl-lysine sites as orange circles (with the residue position defined alongside/within). The size of each acetyl-lysine site is proportional to the increase in acetylation following acetate treatment (quantified as the treated/untreated heavy/light ratio). Pink lines represent a predicted or known interaction (either physical or functional) between two proteins determined using STRING 9.1 from experimental, databases and data-mining information sources with a medium confidence cutoff (0.4).

The extent of acetylation in the ApiAP2 transcription factor family and our results demonstrating that acetylation can alter the DNA-binding recognition of an ApiAP2 DNA-binding domain suggests that acetylation is a mediator of transcriptional regulation beyond histones in *P. falciparum*. We were therefore interested in identifying factors that might influence the acetylation state throughout the intraerythrocytic developmental cycle (IDC). Knowing that the activity of acetyltransferases is dependent upon the presence of acetyl-CoA, we hypothesized that changes in the abundance and availability of this metabolite would influence acetylation. By quantifying the level of acetyl-CoA in parasite cultures throughout blood-stage development (Fig. 6A), we observed a periodic profile for the acetyl-CoA pool, increasing between 3- to 4-fold from a minimum at 5 hours post-invasion to a maximum at 30 hours post-invasion (after which the decrease in acetyl-CoA abundance diverges from the increased biomass of the parasite between 30–48 hours post invasion). This suggests that acetyltransferase activity may be sensitive to the stage-dependent availability of acetyl-CoA, thereby influencing protein acetylation and protein function during asexual parasite development.

*Plasmodium* parasites can generate acetyl-CoA from glucose via the branched-chain keto-dehydrogenase complex (BCKDH) or directly from acetate via acetyl-CoA synthetase (ACS)^31^,^32^. Despite the majority of acetyl-CoA being generated via glycolysis, the presence of acetyl-CoA synthetase presented us with an experimental opportunity to rapidly alter the intracellular pool of acetyl-CoA with minimal changes to overall metabolism (Fig. 6B). Exogenous acetate is rapidly converted to acetyl-CoA^31^ and increases the intracellular acetyl-CoA pool in a concentration dependent manner (Fig. 6C). Here we used isoleucine SILAC-based quantitative proteomics to measure the relative differences in lysine acetylation following a two-fold change in the intracellular acetyl-CoA pool that occurred in response to exogenous acetate supplementation (5 mM for 3 hours at trophozoite stage; heavy = acetate pulse, light = untreated). Relative measurements of the acetylome between the acetate-pulsed and untreated parasites revealed 96 peptides with greater than two-fold difference in abundance (8.4% of the total quantified acetylated peptides at a ≤1% FDR; Supplemental Table 9). Acetate treatment increased acetylation on proteins that were mainly ribosomal, acetyltransferases, and transcription-associated proteins (Fig. 6D). Proteins that underwent increased acetylation following acetate exposure were grouped into functional and/or physical interaction partners, as predicted by a STRING (v9.1) network analysis^33^. Two main nodes were observable, one centered on the acetyltransferases, chromatin/histone proteins and transcription factors, and the second node encompassing ribosomal proteins (Fig. 6D).

Several acetyl-lysine sites that were elevated under acetate exposure were also previously identified as elevated under TSA treatment (Fig. 6D; green sites). In particular, the acetyl-lysines present on the acetyltransferases are regulated by the class I and II KDACs and are also sensitive to the intracellular acetate/acetyl-CoA balance. Moreover, two proteins putatively involved in the GCN5 complex have multiple acetylated lysines that are sensitive to the acetyl-CoA pool and are deacetylated by the class I and II KDACs (PF3D7_1433400 and PF3D7_1008100)^2^,^4^. Interestingly, acetyl-CoA synthetase itself increases in acetylation at position 455 and 459 under acetate and TSA treatment, suggesting that acetyl-CoA synthetase could be an important control point for regulating the acetyl-CoA/CoA balance of the parasite and hence may play a role in regulating the acetylome.

These findings indicate that changes to acetate/acetyl-CoA metabolism may elicit a specific effect on the acetylation of transcription- and translation-associated proteins and not a generalized response across the whole acetylome. The total protein abundance between acetate treatment and the untreated control was no different for each protein of interest (i.e. a H/L ratio approximating 1.0), which indicates that the changes in acetylation levels observed reflect changes in site occupancy and are not a consequence of altered protein abundance following acetate exposure (Supplemental Table 9), similar to our conclusions for tricostatin A treatment.

Acetyl-CoA availability could direct acetylation either by altering the activity of the parasite KATs or via auto-acetylation, which induces acetylation via a non-enzymatic process. However, we cannot discount the possibility that acetate itself is influencing the acetylation state of the parasite, which would require further dissection of the parasite’s acetate/acetyl-CoA balance to determine.

## Discussion

Here we have expanded the breadth of the known acetylome of *P. falciparum* by seven-fold, demonstrating acetylation to be comparable with phosphorylation and ubiquitinylation as a major post-translational modification in the malaria parasite^34^. The disparate functions of acetylated proteins in *P. falciparum* and their widespread distribution across subcellular compartments highlights the global role lysine acetylation plays in parasite development. Although histone acetylation has been well documented^35^, the extensive acetylation of metabolic enzymes suggests that this PTM contributes to the regulation of metabolism in the malaria parasite as observed in other organisms^3^,^36^. Moreover, the enrichment of acetylation in virulence and transcription-associated proteins suggests a complex layer of regulation in processes central to the coordination of parasite development and propagation. Considering the high prevalence of lysine in the *P. falciparum* proteome (in part due to the AT-rich genome; 11.6% prevalence compared to 7.5% in *S. cerevisiae*^37^,^38^), it is plausible that lysine acetylation may play a more significant role as a regulatory switch in parasite development compared to other eukaryotic organisms.

The malaria parasite has a regimented transcriptional program during the IDC that displays a cascade of gene expression^25^. It has been proposed that this unusual ‘just-in-time’ transcriptional program is likely mediated by members of the ApiAP2 DNA-binding protein family^28^. The DNA binding specificity of individual AP2 domains from these proteins has been described^26^ but the function of most ApiAP2 proteins remain uncharacterized^27^,^39^. Understanding how these DNA-binding proteins are regulated to coordinate the transcriptional cascade has remained elusive. Acetylation is prevalent in the ApiAP2 protein family (62 sites on 16 proteins) and may play an important role in directing the transcriptional program. We show that the loss of the positive charge at a lysine following acetylation leads to the loss of specific DNA binding by the second AP2 DNA-binding domain of the ApiAP2 protein PF3D7_1007700. The loss of specific DNA binding following acetylation is consistent with previous reports of bacterial and mammalian transcription factors and cofactors^40^,^41^. Whether the same effect occurs *in vivo* and how the loss of DNA-binding of one domain (via acetylation) affects the function of the other DNA-binding domains within PF3D7_1007700 remains to be tested. Two hypotheses are plausible; firstly the loss of DNA-binding via acetylation of one AP2 DNA-binding domain may increase transcription factor binding preference for the DNA motifs recognized by the other AP2 domains; alternatively it may decrease transcription factor recruitment by inhibiting one of the three domains required for ApiAP2 DNA-binding.

58 acetyl-lysine sites were identified on ApiAP2 proteins that reside outside of annotated functional domains. It remains unclear what impact these modifications may have (if any) on protein function. Plausible roles for these acetyl-lysine sites could include directing protein localization and modulating protein-complex formation, however until this can be explored further, the functional influence of acetylation at these sites remains a mystery^42^,^43^. Validating the role of lysine acetylation in DNA binding and complex formation by members of the ApiAP2 family will require further experiments *in vivo* and is of interest, but nonetheless technically difficult to pursue, considering the essentiality of several ApiAP2 proteins during the blood stage or their exclusive role in other developmental stages.

The composition of transcription factor complexes and how they are regulated in Apicomplexa are poorly defined, but a recent study found that the acetyltransferase GCN5b and ApiAP2 transcription factors interact to form protein complexes in *T. gondii*^44^. GCN5b was also associated with a ‘core complex’ including the co-activator ADA2 and transcription initiation proteins. Here we identified a similar complement of proteins that are acetylated, and their acetylation site occupancy is sensitive to changes in the acetate/acetyl-CoA balance. These two lines of evidence suggest that acetyltransferases form complexes with transcription initiation proteins, and that acetylation plays a role in regulating the formation of specific complexes and/or recruitment to particular regions of the genome. This hypothesis is consistent with the *in vivo* association of *Pf*GCN5 with the ADA2 coactivator^45^ and may explain how GCN5 is recruited to specific promoters to activate gene expression in *Plasmodium* spp.^46^.

The importance of metabolic fluctuations acting as an upstream signal to influence transcriptional activity is gaining increased recognition^42^. Acetyl-CoA is a desirable signaling metabolite because it is rapidly biosynthesized, is pivotal to central carbon metabolism and is the substrate for lysine acetylation by acetyltransferases. By artificially increasing the intracellular acetyl-CoA pool in the malaria parasite, acetylation of translation- and transcription-related proteins increased. This metabolic response appears to be specific, as the proteins affected are functionally related. The periodic nature of the intracellular acetyl-CoA pool during the IDC indicates that the availability of acetyl-CoA may act as an upstream signal for influencing a transcriptional response, as seen in yeast^47^. The transcriptional program of *P. falciparum* is relatively fixed by comparison to most other eukaryotic organisms, hence metabolic co-regulation of transcription may be more important to fine-tuning the transcriptional program in *P. falciparum*. However, metabolite signaling could be a pronounced transcriptional co-regulator as a consequence of dramatic changes in metabolism between developmental stages. Validation of this hypothesis would require genetic disruption of acetyl-CoA metabolism and would help delineate the influence acetate and acetyl-CoA each have separately on regulating the acetylome. Genetic approaches would also assist in defining the contribution of auto-acetylation to protein regulation in the parasite and how it contributes to the overall acetylome.

Lysine deacetylases have been proposed as attractive antimalarial targets given the importance of histone acetylation to transcription^14^. However, our characterization of the acetylome, in which we demonstrate acetylation of proteins associated with most major cellular processes (e.g. transcription, translation and metabolism), suggests the antimalarial action of KDAC inhibition may be much broader than previously appreciated. Here we have begun to identify the acetyl-lysine targets of the parasite KDACs in order to understand the regulatory role of each class of KDAC and thereby understand their antimalarial action. Using TSA, a broad-specificity inhibitor of class I and II KDACs, we identified the specific acetyl-lysine targets of these enzymes. The transcriptional response induced by TSA treatment previously identified substantial modulation of mRNA transcript abundances as measured by DNA microarray^10^. However, inhibiting deacetylation of histones is generally associated with relaxed chromatin and active transcription, thus it remained unclear how down-regulation of transcription could occur after inhibiting histone deacetylation. Here we find that TSA also inhibits deacetylation of transcription-associated and chromatin-remodeling proteins, which may explain the mechanism behind this down regulation or deregulation of transcription.

Beyond the biological relevance of understanding how transcriptional regulation is mediated in *Plasmodium* parasites, the antimalarial potential of KDAC inhibitors justifies further interrogation of the acetylome. In particular, questions regarding the broader functional significance of the acetylome and how it is regulated demand further exploration. The divergent transcriptional response between closely related KDAC inhibitors suggests that the variable composition of deacetylase multi-protein complexes directs acetyl-lysine specificity and inhibitor susceptibility^10^,^48^. The ability to discriminate between inhibitors that target different KDAC complexes (as opposed to individual KDACs themselves)^49^ provides a means for developing specific antimalarial KDAC inhibitors and a way to unravel how different protein complexes may regulate the parasite acetylome.

## Materials and Methods

### *P. falciparum in vitro* cultivation and stable isotope labeling with amino acids in cell culture (SILAC)

Mycoplasma-free *P. falciparum* NF54, 3D7, IG06, Sir2A^−^ and Sir2B^−^ parasite lines were cultured and synchronized by standard methods^50^,^51^ with the modification that culture flasks were maintained at 37 °C in an atmospherically controlled incubator set at 5% CO_2_, 6% O_2_.

^13^C_6_^15^N_1_ isoleucine was purchased from Cambridge Isotopes and substituted for unlabeled isoleucine in specially formulated RPMI at a concentration of 305 μM. Following the method described by Nirmalan and colleagues we validated that after 72 hours >95% of isoleucine in *P. falciparum* protein extracts was isotopically labeled via LC-MS^21^. The dynamic range of the LC-MS-based SILAC ratio measurement was determined empirically via analysis of mixtures of various ratios of isotopically-labeled and unlabeled protein samples.

### Protein extraction and peptide processing

Synchronous parasite cultures were saponin isolated (0.05%) at the trophozoite stage, washed with ice-cold PBS and extracted with guanidine-based solublization buffer (8M guanidine HCl, 100 mM Tris pH 8, 50 mM DTT). We specifically chose to employ guanidine over the frequently used urea chaotrope during lysis, so as to avoid potential artifacts of lysine carbamylation, which can convolute the bioinformatics of acetylation identification, leading to misidentification. Total protein extracted was quantified via BCA assay (Thermo Fisher) and the total protein amount per experiment was 20–40 mg. Protein was subjected to dissolution, thiol reduction and alkylation, and overnight trypsin digestion according to the FASP methodology^52^. Digest solution was subjected to 10 KDa cutoff membrane ultrafiltration (Microcon YM-10, Millipore) and the filter was washed with 50% acetoniltrile (ACN), 0.1% formic acid (FA). Peptides were separated up-front into ca. 12 fractions via strong cation exchange (Supelco SCX SPE, Sigma) and a small portion of each was taken for ‘unenriched’ nano-UPLC-MS and –MS/MS analysis. The remaining portion of the peptide fractions were enriched for acetylated lysine via anti-acetyl-lysine immunoprecipitation (ImmuneChem) overnight at 4 °C. After five washes in Tris-buffer containing ProteaseMAX, acetylated peptides were eluted with 50% acetoniltrile (ACN), 0.1% formic acid (FA). The final peptide liqueur was concentrated in a speedvac until near dryness. Peptides were desalted using StageTip micro-scale reversed-phase chromatography^53^. For SILAC experiments, infected cultures were prepared as described above but equivalent amounts of the treated and control cell pellets (heavy and light respectively) were pooled prior to protein extraction. *In silico* predictions indicate that trypsin digestion of the entire parasite proteome would result in only 61% of all peptides generated containing an isoleucine residue^21^. This would preclude 39% of trypsin-digested peptides from being quantitated by non-standard ^13^C_6_^15^N_1_ isoleucine SILAC and prevent biologically-significant results from being detected.

### High resolution nano-UPLC-MS characterization of acetylated peptides

Enriched and unenriched peptide fractions were subjected to analysis by accurate mass high resolution reversed-phase nano-UPLC-MS and MS/MS performed on an Easy-nLC Ultra 1000 nanoflow capillary UPLC system (ThermoFisher Scientific, San Jose, CA) coupled to a VelosPro-Orbitrap Elite hybrid mass spectrometer (ThermoFisher Scientific) using a Flex ion source (Proxeon, Odense, Denmark). Separation and data acquisition were achieved via methods previously described^54^. The resulting LC-MS/MS data were processed into peak-list files (mgf) using ProteomeDiscoverer (v. 1.4/2.0, ThermoFisher), and then searched against a concatenated database consisting of the proteomes of *Plasmodium falciparum* (PlasmoDB v. 9.2) and human erythrocytes (derived from^55^) using the Mascot search engine (v. 2.4, Matrix Science) within the ProteomeDiscoverer framework, allowing for an initial mass error of 6 ppm for precursor and 1.2 Da for fragment ion species, ≤3 missed trypsin cleavages, carbamidomethylation of cysteine as a fixed modification, with methionine oxidation and N-terminal protein and lysine acetylation as variable modifications. A Mascot Quantification setting allowing for heavy isoleucine (+7Da) was specified to allow for spectral match assignment to heavy peptides. SILAC ratio quantification was achieved through the use of a Precursor Ions Quantifier node in ProteomeDiscoverer in which the heavy channel for pairwise heavy/light MS1 ion abundance ratios was set to isoleucine (+7Da), minimum intensity values were substituted for missing quantification channels, and all ratios were globally normalized to the median protein ratio measurement (to adjust for minor variation in SILAC sample pooling). Heavy/light abundance ratios with only one peptide spectral match were removed and (where possible) peptides with a percent variability greater than 60% were removed from further analysis. The percent variation is reported for each heavy/light ratio and was used as a measure of quantitation accuracy. Where the same acetyl-lysine site was measured across multiple peptides, the mean and standard deviation was calculated to determine the final H/L ratio and percent variation. Standard SILAC ratio cutoffs were chosen to identify substantial changes under each experiment (<0.5 and >2) and were validated by the distribution of acetylated peptide with heavy/light abundance ratios (Supplemental Figures 3–6), wherein between 2–9% of acetylated peptides were above the >2 H/L abundance ratio cutoff. In the SILAC experiments with the Sir2A^−^ and Sir2B^−^ parasite lines, both genetically-disrupted lines were cultured using light isoleucine and the wild type line was fully labelled with heavy isoleucine. These values were inverted at the last step to maintain consistency with the other SILAC experiments (e.g. untreated or control = light). For down-regulated acetyl-lysine sites, peptides were collated and where multiple acetylated lysines were detected on the same peptide each site was cross-checked with previously identified sites that underwent increased acetylation. Where a specific lysine residue was quantified as increasing under a particular condition but was detected within a peptide containing multiple acetylation sites that underwent decreased acetylation this site was removed from the analysis of decreased acetylation, consistent with the notion that the redundant site is increasing in acetylation occupancy relative to the surrounding acetyl-lysine sites. Aggregate search results from ProteomeDiscoverer were collated, consolidated, and subject to PTM site assignment and Ascore^56^ PTM localization confidence calculations using Scaffold and ScaffoldPTM (Proteome Software, Portland OR), as previously described^54^ and the peptide and protein false discovery rate was thresholded at ≤1%. Peptide spectral matches and MS1 traces displaying SILAC H and L pairs for acetylated peptides shown to change in response to TSA or acetate exposure in our study were subject to manual inspection and validation using Scaffold, ProteomeDiscoverer, and the original tandem mass spectra acquired in profile mode using Xcalibur software (ThermoFisher). For rigorous specificity, peptides with ambiguous origin (where they matched to both human erythrocyte and *P. falciparum* proteins) were designated human in origin and removed from further analysis. All raw LC-MS data files have been made publicly available as project ID# 1003 in the Chorus public repository (chorusproject.org). They can be downloaded directly via the following link: https://chorusproject.org/anonymous/download/experiment/6334040711402450702.

To confirm that the observed changes in acetylation following TSA and acetate treatment were changes in site occupancy and not a consequence of changes in the total protein pool, the non-acetylated (unmodified) peptides for each protein of interest (i.e. possessing an acetyl-lysine site that had a H/L ratio >2) were collated and the median H/L ratio determined. The median H/L ratio of the non-acetylated peptides represents the relative protein abundance between the two samples. For the acetate pulse experiment 71 out of 71 proteins could be quantitated with a median H/L ratio 0.96 ± 0.14 (SD), which indicated that the abundance of each protein was unchanged following acetate treatment (Supplemental Table 9, Table 7). TSA treatment also resulted in few changes to total protein abundance, with the median H/L ratio across the 21 proteins of interest approximating to 1 (Supplemental Table 6, Table 7), confirming that the changes in acetylation were genuine changes in site occupancy. The only exceptions to this conclusion were for 3 proteins: a protein of unknown function (PF3D7_1124300; Ac-K H/L ratio = 3.25, total protein H/L ratio = 7.1), one serine repeat antigen (PF3D7_0207900; Ac-K H/L ratio = 100, total protein H/L ratio = 74) and a zinc-finger protein (PF3D7_1134600; Ac-K H/L ratio = 2.3, total protein H/L ratio = 2.4). While these 3 proteins which showed acetylated peptide deviations related to changes in protein abundance, contrary our other observations in this study, may play important roles relevant to the influence of TSA treatment on *P. falciparum,* further exploration of them was beyond the scope of this study and thus they were excluded from further analysis.

### Protein acetylation data analysis

Gene Ontology (GO) data from PlasmoDB 9.0 were used for enrichment analysis. After removing pseudo-genes and proteins without lysines and expanding the GO annotations to include every implied term via the “is_a” relationship, we obtained 119636 GO terms, contained in 4408 proteins. GO terms without any acetylated proteins were removed, leaving 1692 GO terms that were tested for enrichment relative to their adjusted background lysine and acetylated lysine counts. To reduce redundancy in the p-values associated with dependent GO terms, two filters were implemented. First, a GO term is removed if it is implied by a higher-ranking GO term via an “is_a” relationship. Second, a GO term is removed if it implies a higher-ranking GO term via an “is_a” relationship. GO terms were ranked by p-value, with smaller p-values ranking higher. After filtering only 534 GO terms remained and the q-values and E-values were computed for the last set of p-values.

Domains were predicted using HMMER 3.1b1 and the Pfam version 27 HMM database. Three different methods were considered for the domain enrichments, the Standard Pfam (uses expert-curated thresholds), domain stratified q-values (with a threshold of 4e-4, as described in^57^) and tiered stratified q-values (with a per-tier threshold of 1e-4). GO term and domain enrichment was determined using a hypergeometric p-value defined as -


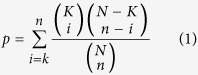


where N is the population size, K is the number of successes, *n* is the number of draws, and *k* is the number of successes among the draws. For domain enrichment N was the number of lysines that fell in domains that belonged to families that contained acetylated lysines in the data and K was the number of these lysines that were acetylated in the data. For each domain family tested, *n* was the number of lysines that every domain instance contained and *k* was the number of these lysines that were acetylated in the data. For GO term enrichment, N was the number of proteins that contained GO terms which annotated at least one protein with acetylation in this data, and K was the number of those proteins that contained at least on acetylated lysine. For each GO term tested, *n* was the number of proteins that it annotated and *k* was the number of those proteins that contained at least one acetylated lysine. Enrichment q-values, which give the proportion of false positives among the significant enrichments, were computed from enrichment p-values^58^. Enrichment E-values, which give the number of false positive significant enrichments, were computed from q-values as the q-value times the number of significant enrichments at that threshold. Raw domain data can be downloaded at http://viiia.org/dssApi/?l=en-us and the code used for domain predictions can be accessed at http://viiia.org/domStratStats/?l=en-us.

Acetyl-lysine motif analysis was performed and presented using the ice Logo motif builder^59^ using a p-value of 0.05. Protein network analysis was performed to represent the functional context of acetylated proteins that underwent changes during each experiment. The STRING9.1 database was used to identify predicted or known protein interactions using experimental, database and text mining information sources with a medium cutoff (0.4)^33^. Associations are derived from either transferring associations between organisms or prediction algorithms that are based on analyzing genomic information. Each information source is benchmarked independently and a combined score is calculated. The score indicates the level of confidence in the association. Most information sources are not compatible with *P. falciparum* (e.g. gene fusion and co-occurrence), so a medium level cutoff was selected to counter the limited number of information sources while minimizing false-positive interactions/associations. Predicted protein interactions/associations are defined as either direct (physical) or indirect (functional) and predictions from each information source was merged into a single predicted/known protein interaction and represented as a single pink line between proteins in Figs 4B and 6D. Histone acetylation conservation was determined using the previous sites reported in *P. falciparum*^4^,^16^ and comparing the merged dataset with the histone acetylation sites present in *H. sapiens*, *S. cerevisiae*, *D. melanogaster* and *M. musculus* by using the CPLM database^17^.

### Cloning and purification of *P. falciparum* AP2 domains for Protein Binding Microarrays

Mutant versions of GST-PF3D7_1007700D2ext, previously referenced as GST-PF10_0075D2^26^ were generated using mutagenic primers and the QuikChange Lightning site-directed mutagenesis kit from Agilent. The wild type and mutant GST-fusions were then purified from *E. coli* bacterial lysates as previously described^26^. The purity of each protein was estimated by Coomassie blue stained SDS-PAGE gels and Western-blots probed with an anti-GST antibody (Clontech). Protein yields were determined based on absorbance at 260nm and specific molar extinction coefficients. The protein binding microarrays (PBMs) were performed in parallel for the wild type and mutant versions of GST-PF3D7_1007700-D2 as previously described^26^. An enrichment score cut-off of 0.45 was used to distinguish high affinity binding data from low affinity and non-specific binding. Two independent replicates were performed for all PBMs.

### Intracellular acetyl-CoA detection

For metabolite LC-MS analyses, samples were extracted and analyzed on an Exactive Orbitrap mass spectrometer as previously described^31^,^60^. For acetyl-CoA profiling across the IDC, tightly synchronized parasite cultures (10% parasitemia; IG06) were extracted in parallel with an uninfected erythrocyte culture (control using the same blood in which the parasites were grown). The relative change in the acetyl-CoA pool was determined as the total ion count for each time point normalized to the uninfected erythrocyte acetyl-CoA signal. These data were originally published in^60^. The shortened IDC of IG06 was normalized to a standard 48 hour for a broader comparison. The concentration-dependent effect of acetate on the intracellular acetyl-CoA pool was determined by incubating >95% magnetically-enriched infected erythrocytes in RPMI 1640 media containing varying concentrations of acetate (pH 7.4) for 1 hour at 37 °C. Metabolite extracts were subsequently generated and analyzed as described above.

## Additional Information

**How to cite this article**: Cobbold, S. A. *et al.* Proteome-wide analysis reveals widespread lysine acetylation of major protein complexes in the malaria parasite. *Sci. Rep.* **6**, 19722; doi: 10.1038/srep19722 (2016).

## Supplementary Material

Supplemental Figures

Supplemental Table 1

Supplemental Table 2-4

Supplemental Table 5

Supplemental Table 6

Supplemental Table 7

Supplemental Table 8

Supplemental Table 9

## Figures and Tables

**Table 1:** Increased acetylation of specific lysine residues in parasite lines with disrupted copies of either Sir2A or Sir2B.

Gene ID	Product Description	H/L Ratio	% Variability	Ac-K Position
∆Sir2A				
PF3D7_1120100	Phosphoglycerate mutase (PGM1)	≥10	0	43*
PF3D7_0821100	Protein kinase 1 (PK1)	≥10	0	349
PF3D7_1311500	26S protease regulatory subunit 7 (RPT1)	4.69	4.5	10
PF3D7_1105000	Histone H4	2.07	48.8	80
PF3D7_0919900	regulator of chromosome condensation, putative	2.04	25.5	1300, 1303
∆Sir2B				
PF3D7_0804900	GTPase-activating protein	≥10	0	220, 246
PF3D7_1107300	polyadenylate-binding protein-interacting protein 1	≥10	0	1403, 1421
PF3D7_1120100	phosphoglycerate mutase (PGM1)	≥10	0	43*
PF3D7_1357800	TCP-1/cpn60 chaperonin family	≥10	0	54
PF3D7_0820700	2-oxoglutarate dehydrogenase E1 component	2.89	1.6	462, 469

^*^Denotes that the same site was elevated (>2 H/L ratio) in both Sir2A and Sir2B genetically disrupted parasite lines.
