# Supplementary material for: Proteome-wide analysis reveals widespread lysine acetylation of major protein complexes in the malaria parasite
Source: Sci Rep. 2016 Jan 27;6:19722. doi: 10.1038/srep19722 (PMC4728587; doi:10.1038/srep19722)

Proteome-wide analysis reveals widespread lysine acetylation of major protein complexes in the malaria parasite

Simon A. Cobbold[a], Joana M. Santos[a,f], Alejandro Ochoa[a], David H. Perlman[a,b,c,d,e], Manuel Llinás[a,b,f*]

[a]Lewis-Sigler Institute for Integrative Genomics, [b]Department of Molecular Biology and [c]Chemistry, and the [d]Collaborative Proteomics and Mass Spectrometry Center, Princeton University, Princeton, NJ 08544

Currently: [f]Department of Biochemistry and Molecular Biology, Department of Chemistry, Center for Malaria Research and Center for Infectious Disease Dynamics, W126 Millennium Science Complex, Pennsylvania State University, State College, PA 16802, USA

[*]Correspondence manuel@psu.edu

Running Title – Regulation of the acetylome in the malaria parasite

Supplemental Figure 1. Acetl-lysine sites on histones and conservation across disparate organisms

Supplemental Figure 2. Protein-protein interaction map of acetylated ribosomal proteins and associated translational proteins. STRING9.1 network analysis was performed with medium confidence (0.4) using database, experiments and neighborhood analysis.

Supplemental Figure 3. Distribution of non-acetylated and acetl-lysine peptides with heavy/light ratios following Tricostatin A treatment

Supplemental Figure 4. Distribution of non-acetylated and acetl-lysine peptides with heavy/light ratios in Sirtuin 2A genetically-disrupted parasites compared to wild type

Supplemental Figure 5. Distribution of non-acetylated and acetl-lysine peptides with heavy/light ratios in Sirtuin 2B genetically-disrupted parasites compared to wild type

Supplemental Figure 6. Distribution of non-acetylated and acetl-lysine peptides with heavy/light ratios following acetate treatment

Supplemental Dataset 1. Complete list of characterized sites of Acetyl-lysine

Supplemental Dataset 2. Comparison of acetyl lysine sites detected in Miao et al. 2013

Supplemental Dataset 3. GO term and domain enrichment analysis

Supplemental Dataset 4. Exported/Host Acetyl-lysine protein list

Supplemental Dataset 5. Summary of each SILAC experiment with >2 and <0.5 H/L pairs

Supplemental Dataset 6. Complete List of TSA SILAC H/L pairs

Supplemental Dataset 7. Complete List of Sir2A SILAC H/L pairs

Supplemental Dataset 8. Complete List of Sir2B SILAC H/L pairs

Supplemental Dataset 9. Complete List of acetate pulse SILAC H/L pairs

Supplemental Figure 1A

29 sites from Trelle 2009 + 5 from Miao 2013 (29/34 sites) including – 24 new sites

K = previously identified site
K = novel site
K = missed site

H2A.Z

MEVPGKVIGGKVGGKVGGKVLGLGKGGKGKTGSGKTKKAPLSRASRAGLQFPVGRV
HRMLKSRISSDGRVGSTAAVYAAAILEYLTAEVLELAGNATKDLKVKRITPRHLQLAIRG
DEELDTLIKATIAGGGVIPHIHKALMNKVPLPPTAQKKPKKN

Ac-K Sites Detected– 6,11,15,19,25,28,30,35,139,144,153,154
Ac-K Sites Missed – 37

H3

MARTKQTARKSTAGKAPRKQLASKAARKSAPISAGIKKPHRYRPGTVALREIRRYQKS
TDLLIRKLPFQRLVREIAQDYKTDLRFQSSAVMALQEAAEAYLVGLFEDTNLCAIHAKRV
TIMPKDIQLARRIRGERS

Ac-K Sites Detected – 10,15,19,24,28,37,38,80,123
Ac-K Sites Missed - 57

H2A

MSAKGKTGRKKASKGTSNSAKAGLQFPVGRIGRYLKKGKYAKRVGAGAPVYLAAVLE
YLCAEILELAGNAARDNKKSRITPRHIQLAVRNDEELNKFLAGVTFASGGVLPNIHNVLL
PKKSQLKAGTANQDY

Ac-K Sites Detected – 124
Ac-K Sites Missed – 4,6

H3.3

MARTKQTARKSTGGKAPRKQLASKAARKSAPVSTGIKKPHRYRPGTVALREIRKFQKS
TDLLIRKLPFQRLVREIAQEYKTDLRFQSQAVLALQEAAEAYLVGLFEDTNLCAIHAKRV
TIMPKDIQLARRIRGERS

Ac-K Sites Detected – 10,15,19,24,28,37,38,80,123

CenH3

MVRTKKNIPNHNPLNAFNRDKSFKTNKTLPNRTVHHGISSKTTNINRPSVNRGGINEV
AQKNLHRTNIRKPHRYRPGVLALKEIRAYQASTQLLIPKIPFVRVVKEITRLFELPDEQFR
YTPEALLALQTASEAYLVSLFEDAYLCSLHANRVTLMPKDIHLARRIRGRD

Ac-K Sites Detected – 24,27,41,61

H4

MSGRGKGGKGLGKGGAKRHRKILRDNIQGITKPAIRRLARRGGVKRISGLIYEEIRGVLK
VFLENVIKDSIMYTEHAKRKTVTAMDIVYSLKRQGRTLYGFGG

Ac-K Sites Detected – 6,9,13,17,32,80
Ac-K Sites Missed - 21

H2B.Z

MSGKGPAQKSQAAKKTAGKTLGPRHKRKRRTESFSLYIFKVLKQVHPETGVTKKSMNI
MNSFINDIFDRLVTEATRLIRYNKKRTLSSREIQTAVRLLLPGELSKHAVSEGTKAVTKYT
TSAA

Ac-K Sites Detected – 4,9,14,15,19

H2B

MVSKKPAKAKKTGTGPDGKKKRKKSRYDSYGLYIFKVLKQVHPDTGISRKSMNIMNSF
LVDTFEKIATEASRLCKYTRRDTLSSREIQTAIRLVLPGELAKHAVSEGTKAVTKFTSK

Ac-K Sites Detected – 4,8,11,65,75,101,113

Supplemental Figure 1B

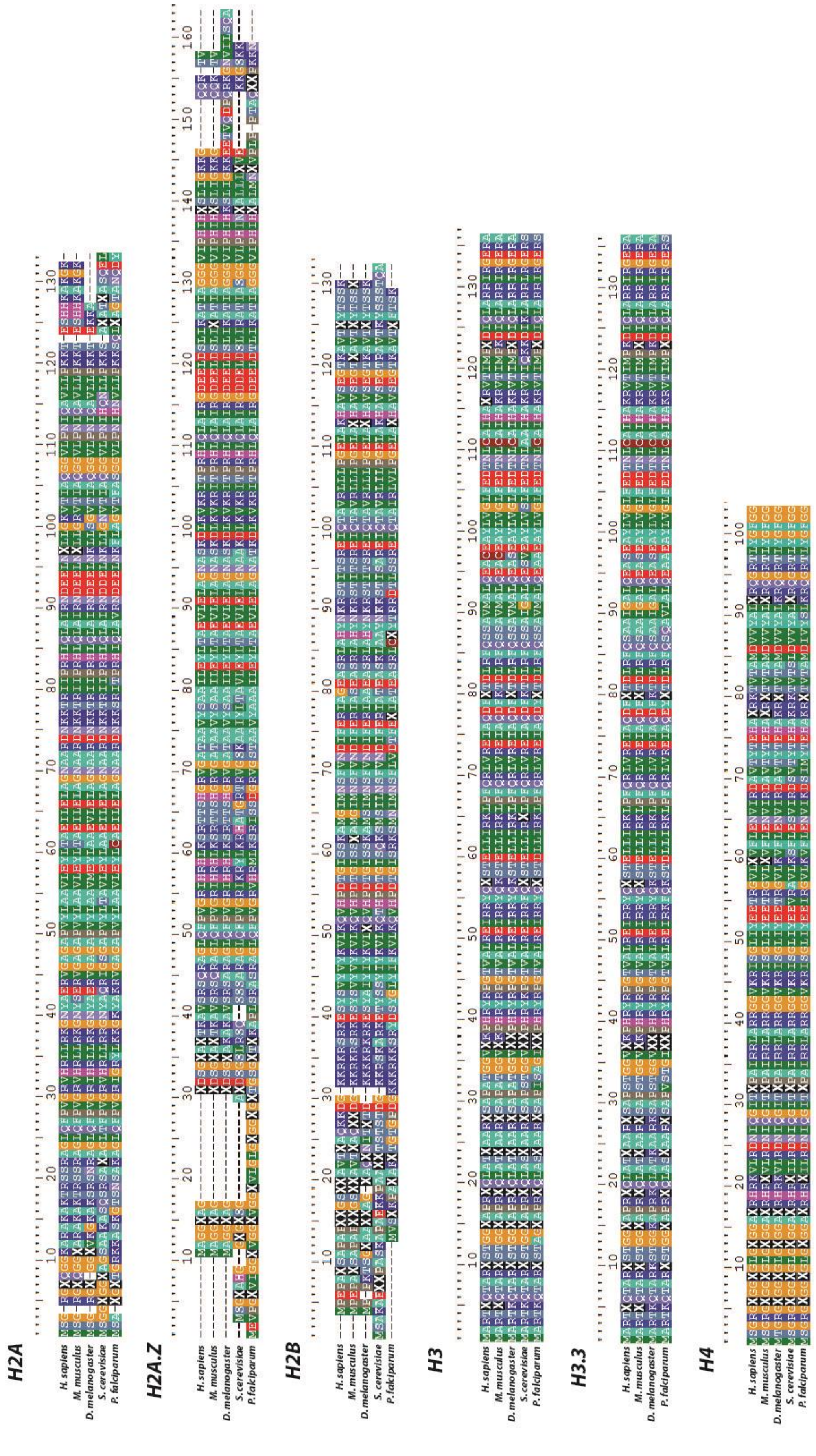

Supplemental Figure 2

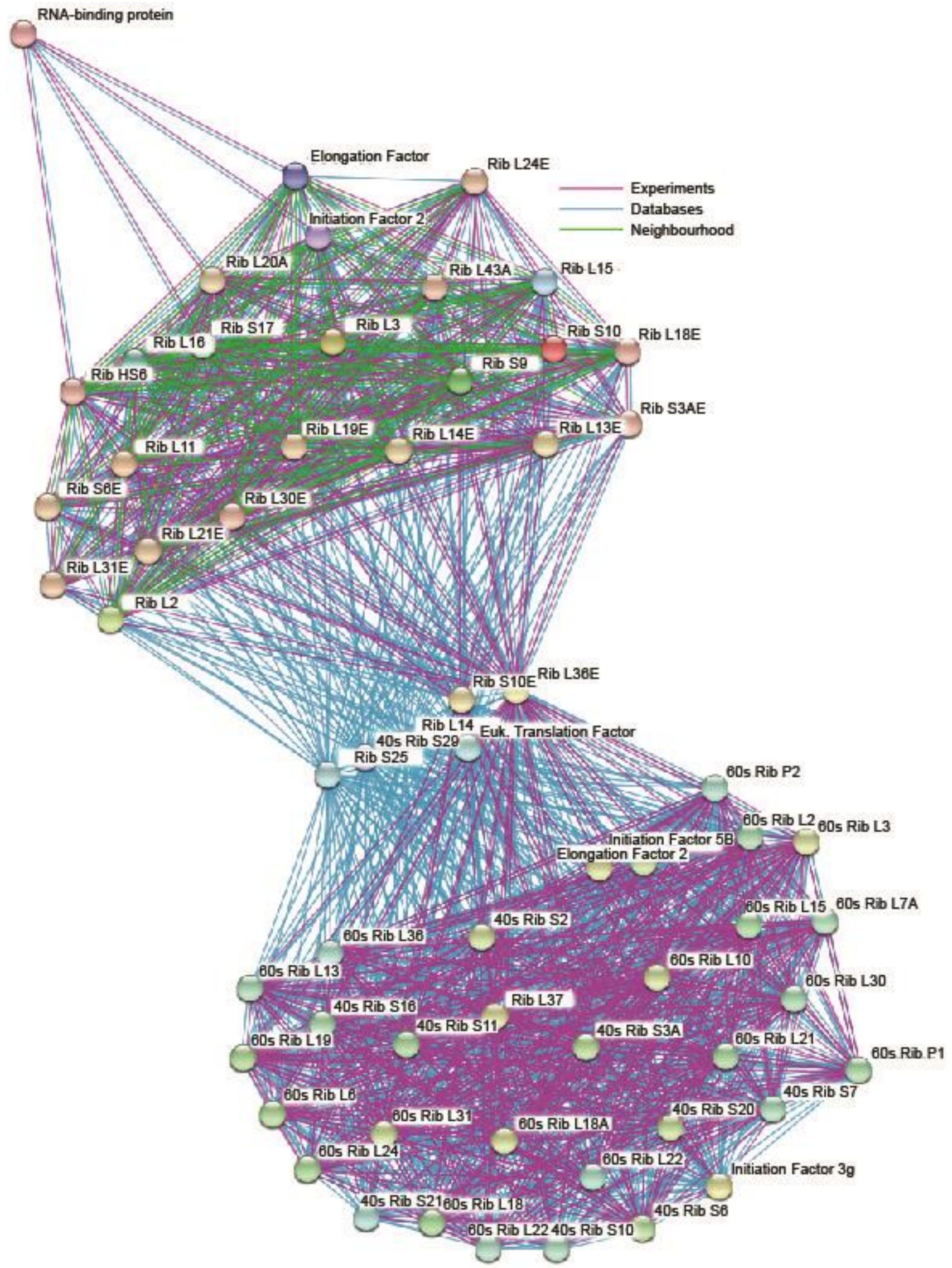

Supplemental Figure 3

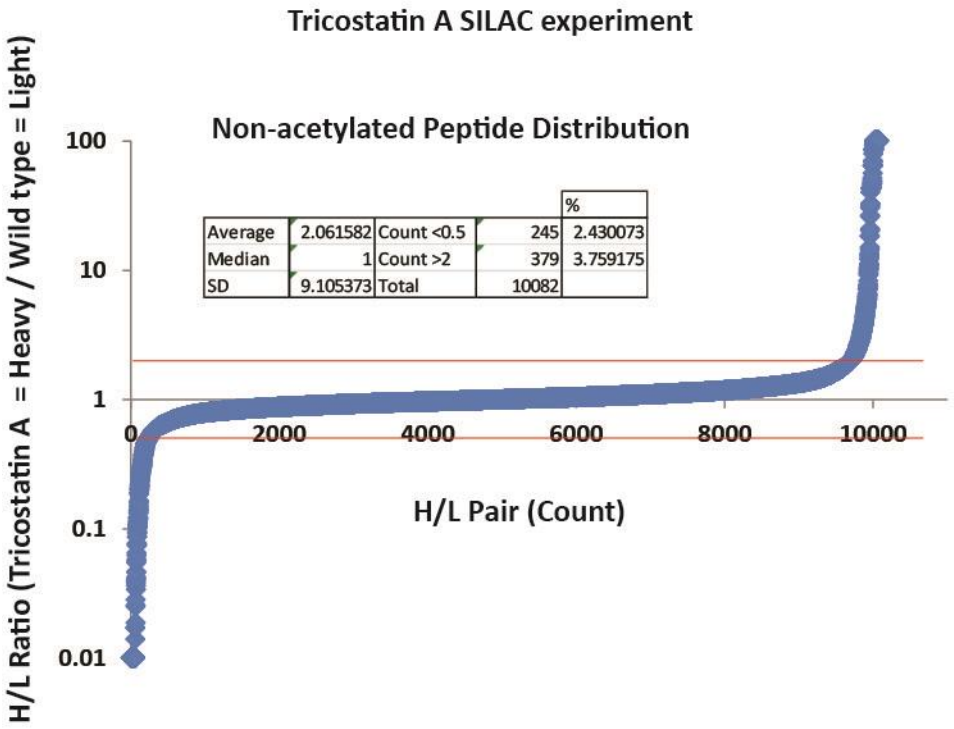

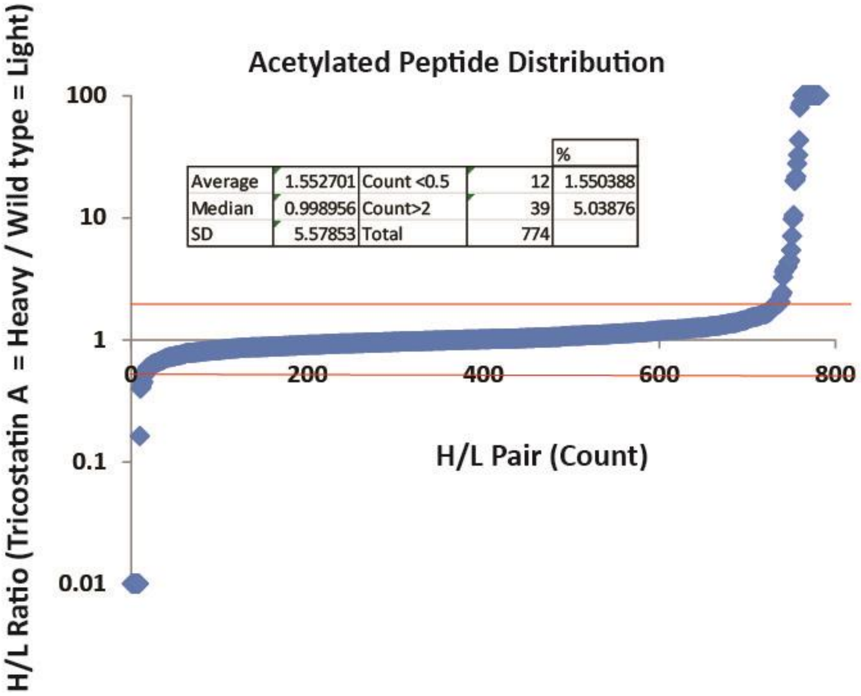

Supplemental Figure 4

Sir2A$^-$ SILAC experiment

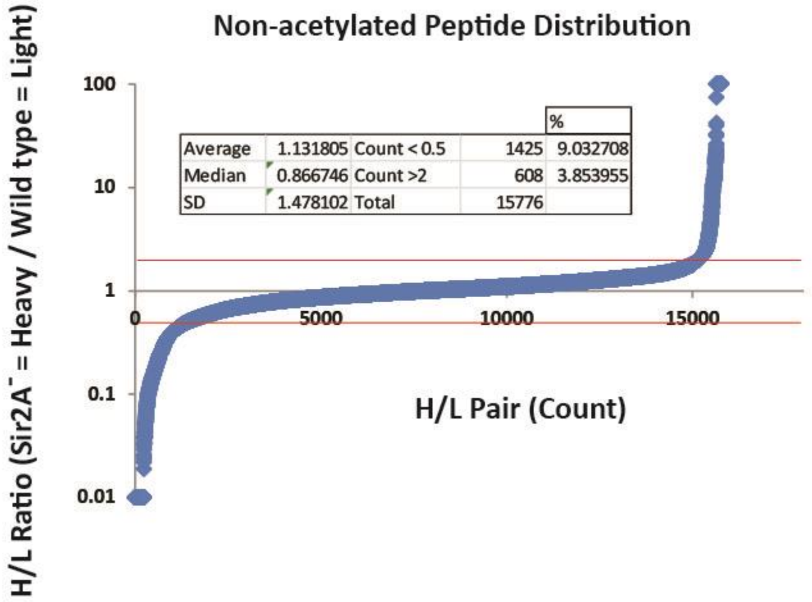

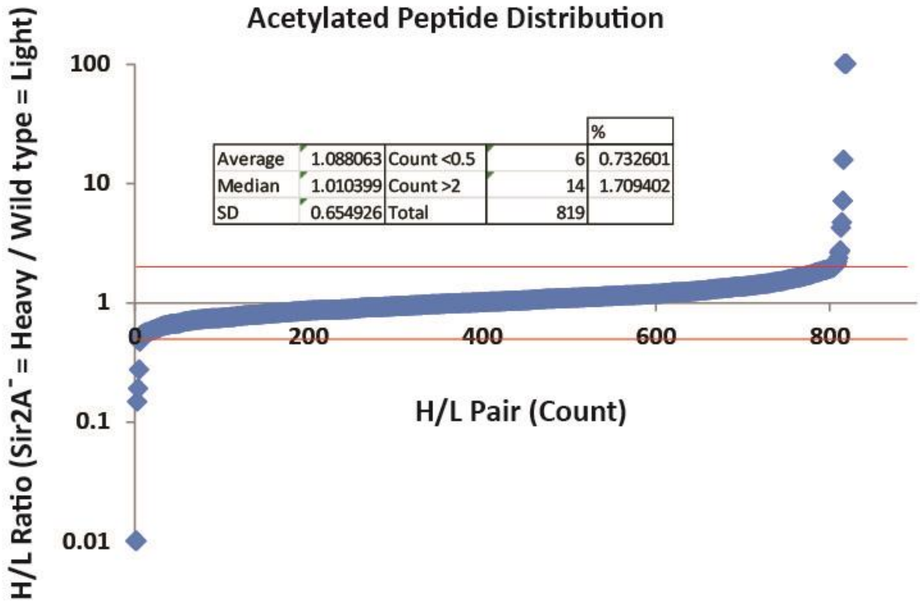

Supplemental Figure 5

Sir2B⁻ SILAC experiment

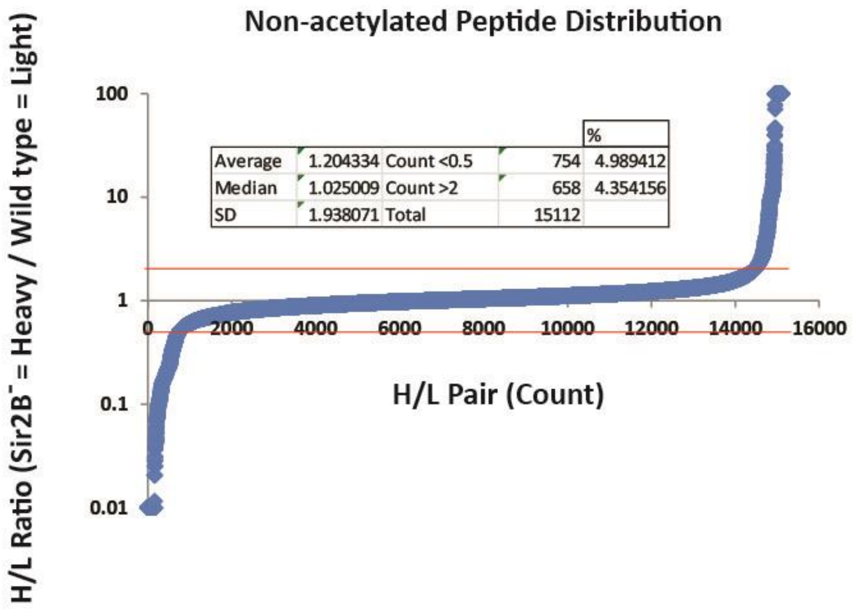

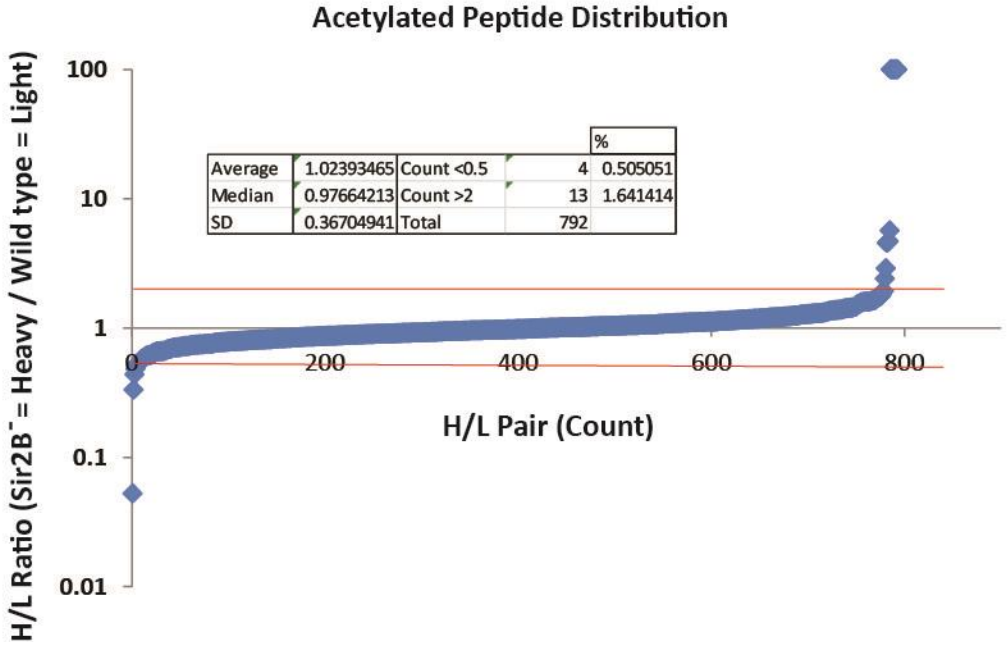

Supplemental Figure 6

Acetate Pulse SILAC experiment

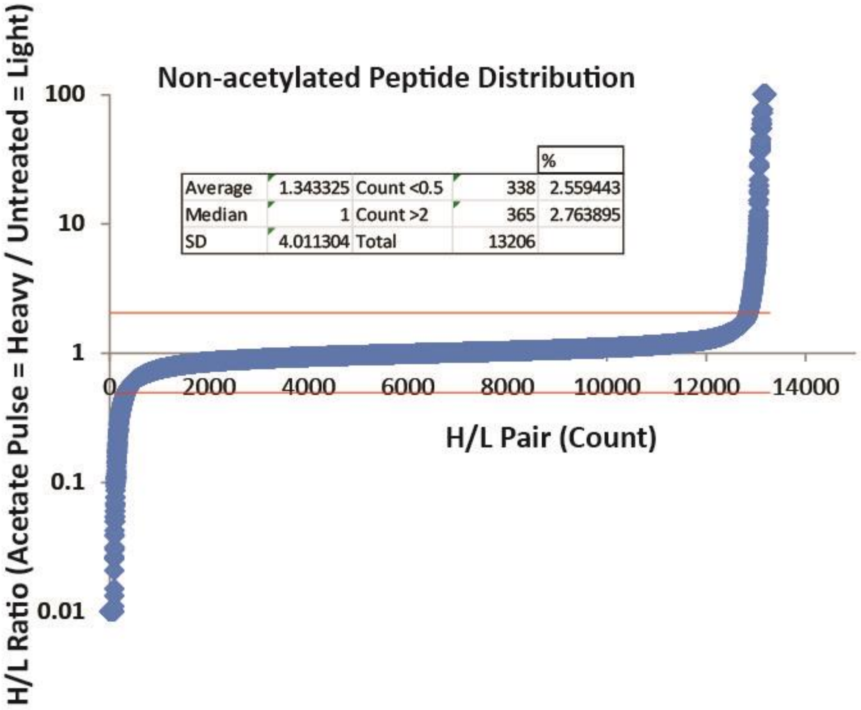

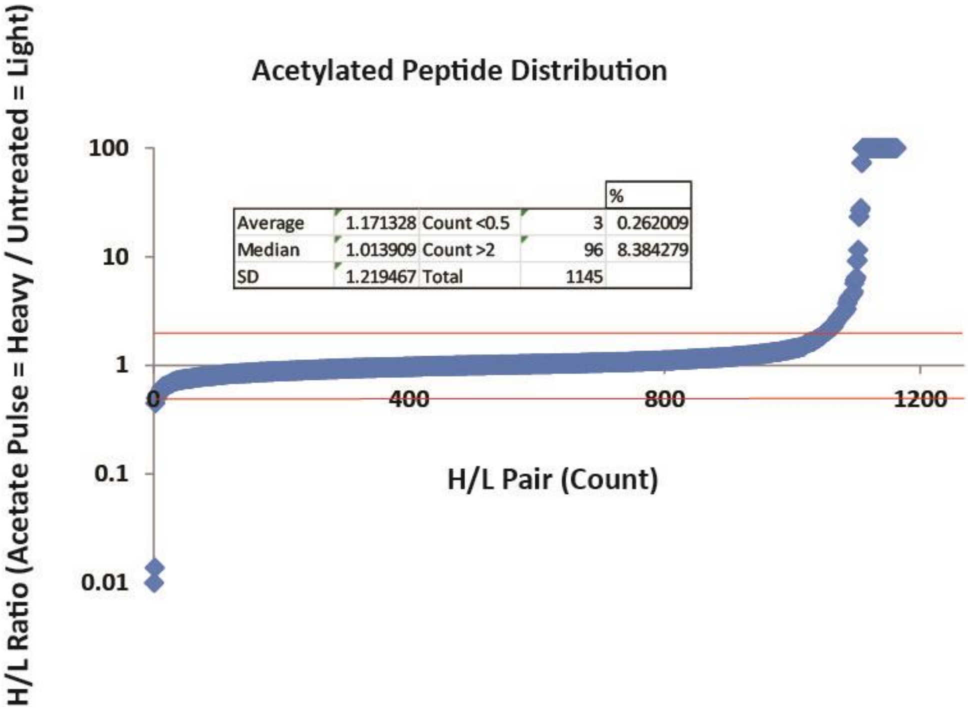

Supplement: Supplemental Figures [file srep19722-s1.pdf]
